# Effect of training community health workers and their interventions on cardiovascular disease risk factors among adults in Morogoro, Tanzania: study protocol for a cluster randomized controlled trial

**DOI:** 10.1186/s13063-018-2924-9

**Published:** 2018-10-11

**Authors:** Alfa J Muhihi, David P Urassa, Rose N M Mpembeni, Germana H Leyna, Bruno F Sunguya, Deodatus Kakoko, Anna Tengia Kessy, Marina A Njelekela

**Affiliations:** 1 Department of Community Health, Muhimbili University of Health and Allied Sciences, United Nations Road, Upanga, Dar es Salaam, Tanzania; 2 Management and Development for Health, Plot # 802, Mwai Kibaki Road, Mikocheni, Dar es Salaam, Tanzania; 3 Department of Epidemiology and Biostatistics, Muhimbili University of Health and Allied Sciences, United Nations Road, Upanga, Dar es Salaam, Tanzania; 4 Department of Behavioral Sciences, Muhimbili University of Health and Allied Sciences, United Nations Road, Upanga, Dar es Salaam, Tanzania; 5 Department of Physiology, Muhimbili University of Health and Allied Sciences, United Nations Road, Upanga, Dar es Salaam, Tanzania; 6 Deloitte Consulting Limited, Aris House, Plot # 152, Haile Selassie Road, Oysterbay, Dar es Salaam, Tanzania

**Keywords:** Cardiovascular diseases, Cardiovascular disease risk factors, Community health worker, Health education, Healthy lifestyle promotion

## Abstract

**Background:**

Cardiovascular diseases (CVDs) increasingly contribute to morbidity and mortality in Tanzania. Public knowledge about CVD risk factors is important for the primary prevention of CVDs and can be improved through community-based interventions delivered by community health workers (CHWs). However, evidence of the utility of CHWs in improving knowledge and CVD risk factors profile is lacking in Tanzania. This study aims at assessing the effect of training CHWs and their CVD-specific interventions for reduction of hypertension and other CVD risk factors among adults in Morogoro, Tanzania.

**Methods:**

This study will use a mixed-methods design with both quantitative and qualitative approaches. A baseline quantitative survey will be conducted to assess knowledge, prevalence, and determinants of CVD risk factors in a random sample of 2950 adults aged 25–64 years. A cluster randomized controlled design with pre-test will be used to assess the effects of CVD-specific interventions delivered by CHWs on reduction of blood pressure and proportion of other CVD risk factors among 516 adults with raised blood pressure from 12 randomly selected villages in Morogoro, Tanzania. Focus group discussion (FGDs) will be conducted at the end of the intervention to assess perceived quality and acceptability of CVD-specific interventions delivered by CHWs.

The intervention will consist of a five-day CVD-specific training to CHWs from villages randomized to the intervention. Trained CHWs will then provide home health education and healthy lifestyle promotion for prevention of CVD risk factors, counseling about hypertension screening for early identification, and referral and linkage of individuals with elevated blood pressure to health facilities. Since intensity of the intervention is key to reinforce behavior change, CHWs will visit the participants every month for the first six months, then bi-monthly thereafter up to 12 months. Except for referral of participants with raised blood pressure identified during the baseline survey, control villages will not receive any interventions delivered by CHWs. At the end of the intervention period, an end-line survey will be conducted in both intervention and control villages to evaluate changes in knowledge, blood pressure, and proportion of other CVD risk factors.

**Discussion:**

The results of this study are likely to have positive policy implications for the prevention of CVD risk factors through the use of CHWs in the provision of CVD-specific interventions, especially now that the Tanzanian government is considering implementing and scaling up a nation-wide multitask CHW cadre.

**Trial registration:**

PACTR Registry, PACTR201801002959401. Registered on 10 January 2018.

**Electronic supplementary material:**

The online version of this article (10.1186/s13063-018-2924-9) contains supplementary material, which is available to authorized users.

## Background

In 2015, cardiovascular diseases (CVDs) accounted for an estimated 17.7 million deaths (nearly one-third of all global deaths), with more than two-thirds of CVD-related deaths occurring in low- and middle-income countries (LMICs) [1]. CVDs are projected to be the leading cause of death and disability globally, accounting for more than 24 million deaths by 2030 [2]. The increasing burden of CVDs in LMICs is fueled mainly by four modifiable risk factors, namely smoking, excessive alcohol drinking, sedentary life style accompanied by physical inactivity, and unhealthy dietary habits characterized by consumption of high energy-dense foods [3]. Tanzania is not spared by the increasing burden of CVDs as available data indicate rising trends of CVD risk factors [4, 5]. In the 1990s, prevalence of hypertension and diabetes in Tanzania was < 0.5%, while that of overweight and hypercholesterolemia was 5.4% and 7%, respectively [6, 7]. In recent years, CVD risk factors have increase dramatically, especially among young and middle-aged adults [4], contributing to significant morbidity, disability, and mortality [8–10]. Prevalence of hypertension has been reported to be as high as 57% with that of severe hypertension at 30%. Obesity, diabetes, and metabolic syndrome are 23%, 6%, and 38%, respectively [5]. The annual incidence rate of stroke has been reported to be 94.5 per 100,000 people in rural settings and 107.9 per 100,000 people in urban settings [9].

CVD-related mortality has declined significantly in high-income countries (HICs), largely because of effective interventions that focused on reduction of population risk factors such as high blood pressure, smoking, and hypercholesterolemia [11–13]. In Finland, such interventions accounted for 53–72% of the decline in coronary heart disease (CHD) mortality between 1982 and 1997, while improved treatments explained only 23% [11]. In England and Wales, the reduction of population risk factors accounted for 58% of the decline in CVD mortality between 1981 and 2000 [14]. Similarly, the decline in CHD mortality in the Netherlands between 1997 and 2007 was attributable to decreases in risk factors and treatment of heart failure and chronic angina in the community [13]. On the other hand, CVD-related mortality continues to rise in most LMICs causing premature and preventable loss of life [15]. Despite the rising burden, there is a lack of appropriate responsive interventions for the primary prevention of known risk factors. Additionally, the health systems in most LMICs are weak and unprepared to deal with the double burden of both prevalent infectious diseases and that of rising non-communicable diseases (NCDs). Thus, population-wide measures that are cost-effective, sustainable, and scalable are best buys for the prevention and control of CVDs in LMICs [16].

Community-based interventions implemented by community health workers (CHWs) have already shown promising benefits in reducing hypertension, incidence of diabetes, improved body weight, and changes in behavior related to physical activity, dietary habits, and smoking [17–20]. However, little evidence is available on their utility towards prevention of CVD risk factors in African settings with varying sociocultural and economic developments [21, 22].

In Tanzania, CHWs are defined as members of the community who are chosen by, and accountable to, the community to provide preventive and health promotion services to their community [23]. They act as the link to bridge cultural and language barriers, expanding access and coverage of health services and thus improving the health outcomes of their community. CHWs have been involved in implementing the interventions for maternal, newborn, child, and adolescent health services [24]. This proposed intervention study is timely in Tanzania as the government plans to roll out multi-task CHWs who will be integrated into the health system for sustainability country-wide [25]. This provides an opportunity to use CHWs to address CVD risk factors at a community level and to implement appropriate interventions for primary prevention.

The study will apply the health belief model (HBM), the first theory that exclusively focused on health-related education and health promotion [26]. The HBM has six constructs of perceived susceptibility, perceived severity, perceived benefits of taking a preventive action, perceived barriers, cues to action, and self-efficacy. HBM has been applied to interventions focusing on hypertension in different settings and populations with success [27, 28]. The findings of this study will provide evidence on the effect of the CHWs’ home-delivered intervention for reducing hypertension and other CVD risk factors and will inform the national initiatives for the prevention of CVD risk factors in Tanzania.

## Methods

### Trial design

This study will use a mixed-methods design with both quantitative and qualitative approaches. A baseline quantitative survey will be conducted to assess knowledge, prevalence, and determinants of CVD risk factors in a randomly selected sample of adults aged 25–64 years. Pre- and post-test design will be used to assess changes in knowledge of the CHWs about CVD risk factors following a five-day CVD-specific training. Trained CHWs will then provide home health education and healthy lifestyle promotion for the prevention of CVD risk factors in the selected intervention villages in Morogoro. A cluster randomized controlled design with pre- and post-intervention design will be used to assess the effects of interventions delivered by CHWs on the knowledge and proportion of CVD risk factors. Qualitative techniques will be used to conduct focus group discussion (FGDs) to participants with elevated blood pressure identified during baseline survey to assess their perceptions about quality and acceptability of the CHWs to deliver CVD-specific interventions. FGDs will be conducted at the end of intervention period.

### Trial site

The study will be conducted in the Kilombero and Ulanga districts within the Morogoro region, approximately 450 km south-west of Tanzania’s commercial capital of Dar es Salaam. The two districts have a population of 407,880 and 265,203, respectively [29]. Kilombero and Ulanga have comparable sociodemographic and economic characteristics, with rice farming being the main economic activity.

The Kilombero and Ulanga districts are selected for this intervention study because of the high number of trained CHWs recorded (14 per 10,000 population) [30] and the reports of rising deaths from NCDs [8]. Data from the Ifakara health and demographic surveillance systems (HDSS), which covers an area of 2400 km^2^ across Kilombero and Ulanga districts, indicated an 8% (16–24%) increase in the number of deaths from NCDs over a period of four years in 2003–2007, with CVDs being the most common NCD-related cause of death [8].

### Participants and eligibility criteria

This study will comprise two groups of participants: CHWs and community members.

1. Community health workers

CHWs from villages selected for the intervention will participate in the training and delivery of the intervention in their villages. They will be assessed on their level of knowledge about CVD risk factors before and after the training session as well as at the end of the intervention to assess knowledge decay. Selected CHWs will have a minimum of four years of secondary education and will have received training for becoming a CHW.

2. Community members

Participants for the baseline survey will include adults aged 25–64 years who are residents of the study villages and who will provide a written informed consent. A resident will be defined based on the HDSS definition as an individual who has stayed in a household within the study village for at least four months continuously, regardless of whether s/he has slept in that household a night before the interview [31]. A household will be defined as group of people who are served from the same pot. Exclusion criteria for cross-sectional surveys will include age outside the specified range, non-residents of the study villages, bed-ridden and/or mentally ill members, and the inability or refusal to provide informed consent for participation.

Participants with elevated blood pressure will be eligible and invited to participate in the CHWs’ home-delivered intervention focusing on health education and healthy lifestyle promotion for the prevention of CVD risk factors. An end-line survey will be conducted to all participants in the intervention study. At the end of the intervention period, a sub-group of participants from villages randomized to the intervention will be randomly selected and invited to participate in FGDs, to assess their perceptions on quality and acceptability of the CHWs’ delivered interventions for the prevention of CVD risk factors.

### The intervention

The intervention package will comprise the following: (1) training of CHWs on CVD factors; (2) home health education and healthy lifestyle promotion; and (3) referral and linkage to health facilities. The intervention will be implemented monthly for the first six months, then bi-monthly up to 12 months. Details of the intervention components are described below.

1. Training of CHWs on CVD risk factors

The first component of the intervention is to provide a five-day training to CHWs from the intervention villages on CVD risk factors. The training will be designed and conducted based on the WHO training manual for the prevention of NCDs and healthy lifestyle [32] and the Tanzanian Curriculum for Basic Technician Certificate in Community Health (a curriculum used for the one-year CHW training course) [33]. For the purpose of this training, only CVD-related topics within the Tanzanian curriculum will be covered. The training will include slow-paced lectures, in-class exercises, and practical sessions. Briefly, the training will cover the following:

i. *Definition and overview of major CVDs.* A brief overview of the major CVDs common in Tanzania such as cerebrovascular diseases, coronary heart diseases, hypertensive heart diseases, and heart failure. Will be covered during the training.
ii. *Overview of both modifiable and non-modifiable CVD risk factors.* Modifiable behavioral CVD risk factors to be covered will include smoking, excessive alcohol drinking, sedentary lifestyle, unhealthy dietary habits, psychosocial stress, and socioeconomic status. Non-modifiable risk factors will include age, gender, genetic/family history, and ethnicity/race.
iii. *Obtaining and calculating body measurements.* CHWs will be trained how to measure body weight, height, waist and hip circumferences as well as how to calculate and interpret body mass index (BMI) and waist-to-hip ratio (WHR). They will also receive training on how to measure and interpret blood pressure using an automated upper arm Omron blood pressure monitor and fasting blood glucose using a point-of-care device.
iv. *Screening procedures and interpretation of results.* CHWs will be trained on the concept of screening for CVD risk factors with an emphasis on overweight and obesity (classification and BMI cut-offs for overweight and obesity), hypertension (blood pressure measurement and cut-offs for hypertension), and diabetes mellitus (DM) (fasting blood glucose measurements and cut-offs for DM).
v. *Home health education and healthy lifestyle promotion.* CHWs will be trained on the importance of smoking cessation, reduction of excessive alcohol drinking, healthy dietary habits (such as high consumption of green leafy vegetables and fresh fruits, low salt diet, use of vegetable cooking oil, and use of whole grains), physical activity and exercise as well as regular health check-ups.
vi. *Effective communication for behavior change.* Behavior change is central to the success of this intervention. An experienced behavioral change communication specialist from Muhimbili University of Health and Allied Sciences will train CHWs on effective behavior change communication strategies and skills for healthy lifestyle behavior change.

2. Home health education and healthy lifestyle promotion

Following the training, CHWs in the intervention villages will provide health education to raise awareness about CVD risk factors and promote healthy lifestyles during home visits. The health messages will focus on information pertaining to the deleterious effects of CVD risk factors, maintaining normal/ideal body weight, smoking cessation, reduction of excessive alcohol drinking, reduction of salt intake, increased consumption of fruits and green leafy vegetables, use of vegetable cooking oil, and use of unrefined cereals. CHWs will also review and provide flyers/brochures on CVD risk factors to participants for their reference. CHWs will use motivational and effective communication for behavior change to encourage participants to change unhealthy lifestyle behaviors.

3. Referral and linkage to health facility

In both the intervention and control villages, participants found to have elevated blood pressure and elevated fasting blood glucose during the baseline survey will be given referral notes to the health facility for further evaluation and appropriate management using standard Tanzanian guidelines. For intervention villages, CHWs will conduct routine follow-ups and ask about their attendance to health the facility as they were advised. For those who will have not attended, reasons for non-attendance will be inquired. The intervention package is summarized in Table 1 below.

**Table 1:** Summary of activities and assessments for intervention and control villages

Activity	Interventions and assessments	
	Intervention villages	Control villages
1. Baseline cross-sectional survey a. Sociodemographic characteristics b. Knowledge of CVD risk factors c. Proportion of CVD risk factors	Yes	Yes
2. Pre-training assessment of CHWs on knowledge about CVD risk factors	Yes	–
3. Training of CHWs on interventions for CVD prevention	Yes	–
4. Post-training assessment of CHWs on knowledge about CVD risk factors	Yes	–
5. Monthly CHW home-delivered interventions (M1–M6) a. Home health education b. Healthy lifestyle promotion c. Measurements (BP and anthropometrics)	Yes	–
6. Bi-monthly CHW home-delivered interventions (M7–M12) a. Home health education b. Healthy lifestyle promotion c. Measurements (BP and anthropometrics)	Yes	–
7. Focus group discussions for perceived quality and acceptability of CHWs to deliver CVD-specific interventions	Yes	–
8. Post-intervention assessment of CHWs for knowledge decay	Yes	–
9. End-line cross-sectional survey a. Sociodemographic characteristics b. Knowledge of CVD risk factors c. Proportion of CVD risk factors	Yes	Yes

### Outcome measures

#### Primary outcome

The primary outcome measure is change in mean systolic blood pressure at 12 months in the intervention villages compared to control villages. The study aims to detect a mean change of ≥ 6.5 mmHg in systolic blood pressure (SBP) in the intervention villages compared to control villages.

#### Secondary outcomes

Secondary outcome measures will include: (1) change in mean diastolic blood pressure at 12 months; (2) change from baseline in the proportion of overweight/obesity, smoking, alcohol intake, unhealthy dietary habits, and physical inactivity; (3) change from baseline in the proportion of individuals at high risk for CVD (10-year risk for CVD > 30%); (4) perceptions about quality and acceptability of CVD-specific interventions delivered by CHWs; and (5) CVD knowledge decay among CHWs at 12 months after training.

#### Intervention process measures

The study also proposes to obtain and evaluate the following intervention process measures: (1) knowledge gain among CHWs following the training; (2) proportion of CHWs visits to study participants out of required visits per protocol; (3) proportion of participants with elevated blood pressure during the baseline survey who went to health facilities as per referral note; (4) proportion of participants with high blood pressure initiated on antihypertensive mediation; and (5) CVD knowledge gained by participants following the CHWs intervention.

### Sample size estimation

This will be a two-arm cluster randomized controlled trial using villages as units of randomization. The minimum sample size for the baseline cross-sectional survey is estimated according to the methods proposed in the WHO STEPwise approach to chronic disease risk factors surveillance (STEPS) [34]. The sample size is calculated for 95% confidence intervals (CI) (z = 1.96) on the basis of a 5% margin of error and an estimated prior population prevalence of hypertension of 25.9% from the nation-wide representative STEPS survey in Tanzania [35], a design effect of 1 and an anticipated attrition rate of 20%. Participants will be adults aged 25–64 years and categorized into four age groups for each sex, resulting in eight strata. The resulting sample size is 2950. Participants with elevated blood pressure during the baseline survey will be eligible and invited to participate in the intervention study.

The sample size for the intervention study is calculated to provide an 80% power to detect a ≥ 6.5 mmHg difference in mean SBP between the intervention and control villages at an alpha level of 5%, assuming an attrition rate of 20%, an inter-cluster correlation of 0.03, and a standard deviation (SD) of SBP of 15 mmHg. The resulting sample size is 258 participants per arm of six villages. Thus, the total sample size for intervention study will be 516 participants from 12 villages.

### Sampling procedures

Participants for the baseline cross-sectional survey will be randomly selected from the intervention and control villages, using a multistage cluster random sampling technique. Lists of households in the selected study villages will be obtained from the village/sub-village leaders. From these lists, we will randomly draw a sample of households for each study village. At the level of household, a list of household members aged 25–64 years will be prepared. For households with more than one eligible participant, a simple random procedure using the next birthday rule will deployed to select an eligible respondent for interview.

### Randomization and blinding

**Fig. 1:**
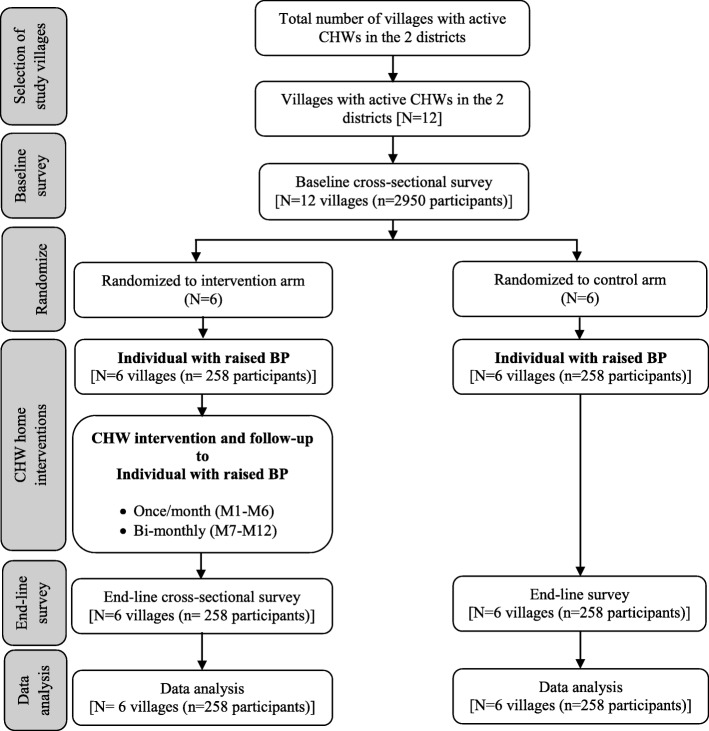
Study design and *flow chart*

The unit of randomization will be the village. A total of 12 villages (six from each district) will be randomly selected from the list of villages with active CHWs. The selected villages from each district will be grouped into two groups of three villages based on population size, type of health facility serving the village, and geographical distance between villages. One group of three villages from each district will be randomly assigned to intervention and another to control. This will ensure that intervention villages are bundled together but far away from control villages, hence minimizing contamination. The final randomization of intervention and control villages will be done by a statistician who is not involved in the implementation of field activities. Randomization of villages to the intervention and control arms will be done by a senior statistician based at Muhimbili University of Health and Allied Science in Dar es Salaam, Tanzania. Assignment of villages to either the intervention or control arm will be done after the baseline survey. Given the nature of the intervention, it is not possible to blind the CHWs who will be involved in the delivery of the intervention. The study design and flow chart are shown in Fig. 1. The SPIRIT checklist is provided in Additional file 1.

### Data collection procedures

#### Pre- and post-training assessments to CHWs

Before starting CHW training, a self-administered questionnaire will be given to all CHWs from the intervention villages to assess their prior knowledge about CVD risk factors. The questionnaire will be adapted from the WHO training manual on NCDs and healthy lifestyle promotion. The tool will assess CHWs’ knowledge on: (1) causes and risk factors for CVDs; (2) warning signs for CVD events; (3) course of action to be taken in case of CVD events; (4) unhealthy dietary habits; and (5) CVD prevention measures. The pre-training assessment will serve as the source for estimation of baseline levels of knowledge for the trainer(s) to understand how much the CHWs already know about CVD risk factors, identify knowledge gaps, and customize training material to address these areas during training. The same tool will be administered after the training sessions to evaluate changes in knowledge of the CHWs. A follow-up assessment will be done at the end of the study (12 months) to all CHWs in the intervention arm to assess knowledge decay over time.

#### Baseline survey

A cross-sectional survey will be conducted in all selected villages at the beginning of the study and before randomization. Trained research assistants will conduct face-to-face interviews in Swahili with selected members of the community using a questionnaire. The questionnaire will be developed in English, translated into Swahili, and back-translated to English to check if it retained its original meaning. Collected data will include sociodemographic characteristics, knowledge on CVD risk factors, modifiable CVD risk behaviors (smoking, alcohol consumption, and dietary habits), and physiological CVD risk factors (elevated blood pressure, overweight, obesity, and elevated fasting blood glucose). Participants found to have elevated blood pressure and elevated fasting blood glucose during the baseline survey will be given referral notes to a health facility for further evaluation and appropriate management using standard Tanzanian guidelines.

#### Follow-up

Participants in the intervention study will be followed up by CHWs at home every month for the first six months, then bi-monthly up to 12 months. In addition to the health education provided during home follow-up visits, CHWs will also monitor and measure blood pressure, adherence to medication (if on medication), and provide further counseling on risk factors and continued healthy lifestyle promotion.

#### End-line survey

An end-line evaluation survey for participants in the intervention study will be conducted by an independent team. Outcome variables of interest assessed during baseline will be re-assessed during end-line to allow for assessment of changes in blood pressure, knowledge, and proportion of CVD risk factors following the CHWs delivered interventions.

#### Focus group discussions

We will conduct FGDs at the end of the intervention to assess the perception of participants about quality and acceptability of CHWs to deliver CVD-specific interventions for prevention of CVD risk factors. FGDs will be conducted for intervention participants and key community members (local political and religious leaders). Each FGD group will consist of 8–10 participants. FGDs will be conducted separately for intervention participants and key community members. An FGD guide with questions related to perceptions about quality and acceptability of the CHWs to deliver CVD-specific interventions will be used to lead the discussions. All FGDs will be audio recorded using an Olympus digital voice recorder.

#### Measurements of social and cardiovascular risk factors

##### Knowledge about CVDs

Knowledge about CVDs will be assessed using a structured questionnaire with variables adopted from other sources [36, 37]. Assessment of knowledge about CVDs will comprise the following knowledge domains: (1) causes and risk factors for CVD; (2) warning signs for CVD events; (3) course of action to be taken in case of CVD events; (4) unhealthy dietary habits; and (5) CVD prevention measures.

##### Blood pressure

Blood pressure will be measured using a standardized digital blood pressure machine (OMRON HEM-712C). Blood pressure will be measured on the left arm with the participant in a seated position. The first reading will be taken after resting for at least 5 min. The second and third readings will be taken halfway and at the end of interview, respectively. An average of three readings will be used in the analysis. Participants with elevated blood pressure will have measurements repeated on the following days to confirm their elevated blood pressure. Hypertension will be defined as average SBP ≥ 140 mmHg and/or DBP ≥ 90 mmHg and/or current treatment with antihypertensive medications in accordance with the Seventh Report of the Joint National Committee on Prevention, Detection, Evaluation and Treatment of High Blood Pressure [38].

##### Blood glucose

Fasting blood glucose will be measured the next day using a Gluco plus glucometer point-of-care device following overnight fasting (at least 8 h of fasting). Elevated fasting blood glucose will be defined as ≥ 126 mg/dL or ≥ 7.0 mmol/L [39].

##### Behavioral risk factors

A modified WHO STEPwise data collection tool for surveillance of non-communicable diseases will be used to gather information on behavioral CVD risk factors including smoking, alcohol drinking, and dietary habits. The WHO STEPwise data collection tool has already been used in Tanzania for the assessment of behavioral CVD risk factors [35]. Questions will probe on current and past smoking as well as current and past alcohol drinking. Dietary assessment will include intakes of fruits, vegetables, salt, use of vegetable cooking oil, and consumption of whole grains.

##### Anthropometrics

Anthropometric measurements will include weight, height, and waist circumference taken using standard procedures. Briefly, all anthropometric measurements will be taken with the participant wearing light clothing and without shoes within the participant’s household compound. Body weight will be taken to the nearest 0.1 kg using a SECA digital scale placed on flat ground. Height will be taken in a standing position with heels perpendicular to the portable stadiometer, measured to the nearest 0.1 cm. BMI will be calculated as body weight divided by height squared (kg/m^2^). Overweight will be defined as BMI ≥ 25 kg/m^2^ but < 30 kg/m^2^ and obesity is defined as BMI ≥ 30 kg/m^2^ [40]. Waist circumference will be measured and recorded to the nearest 0.1 cm using a non-stretchable measuring tape at the mid-point between the lower margin of the last rib in the mid-axillary line and the iliac crest according the WHO guidelines [41]. Abdominal obesity will be defined as waist circumference ≥ 102 cm.

##### Socioeconomic status

Data on ownership of household items such as radio, television, telephone, sofa, refrigerator, bicycle, car, and having working electricity; house ownership, construction materials (floor, walls, and roofing materials); source of fuel for cooking and lighting; source of water supply for home use and drinking; and sanitation facility will be collected [42]. A household wealth index in quintiles will then be generated following descriptions in the Demographic Health Survey toolkit [43].

##### Other data

**Fig. 2:**
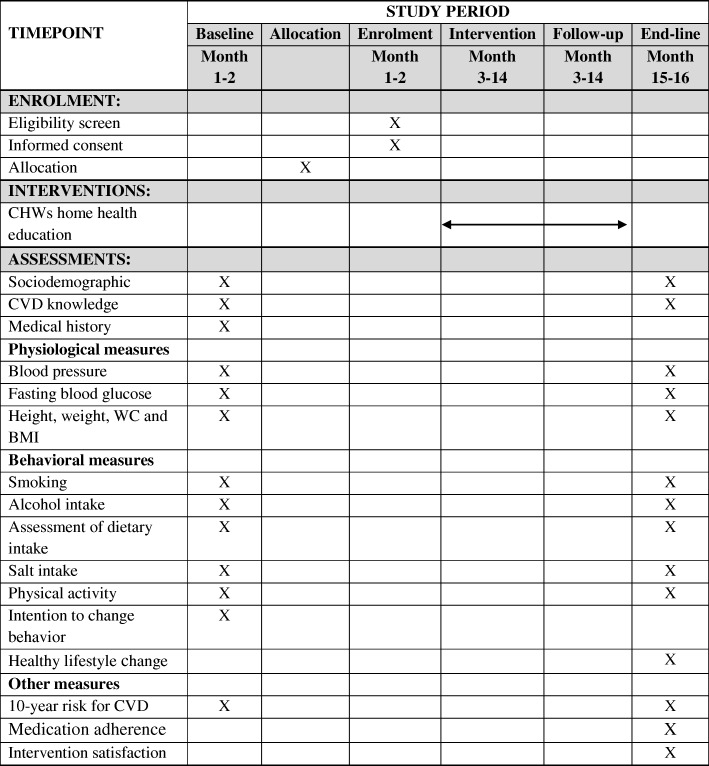
*Schedule* for enrolment, interventions, and assessments

Sociodemographic information including age, gender, marital status, education level, and occupation will be collected. These variables have already been used in population surveys in Tanzania [35, 42]. Age will be collected as a continuous variable. Education will be measured as years spent in school and highest education level attained. Marital status will be grouped into never married, married or living together, divorced or separated, and widowed at the time of data collection. Occupation will be assessed as a categorical variable as none or housewife, employed (public/private), peasant, petty business, and others. Schedule for enrolment, interventions, and assessments for this study are summarized in Fig. 2 below.

### Data analysis

#### Quantitative data

The statistical analyses will be conducted using IBM SPSS statistics version 20, following the Consolidated Standards for Reporting Trials (CONSORT) guidelines for cluster randomized trials [44]. In the initial analysis, baseline characteristics of participants in the intervention study will be compared between intervention and control arms to ensures an approximate balance in the distribution of both measured and unmeasured confounders. Primary analyses will follow the intention-to-treat principle at 12 months, where all participants will be analyzed in their assigned intervention arm regardless of whether they actually received the intervention or not. Participants who refuse/withdraw their consent to continue with study will be excluded from the final analyses.

The primary outcome for the study is continuous mean SBP measured at baseline and at the end of participant follow-up. Change in mean SBP between baseline and end of intervention will be analyzed using individual-level data to assess the effectiveness of CHWs’ interventions for the reduction of blood pressure in the intervention arm compared to control arm. Between-group and within-group differences in mean SBP will be calculated and compared using difference-in-differences (DID) methods [45]. This approach will compare changes in mean SBP between baseline and 12 months for intervention and control villages.

Thus, IMPACT = [Y_i (*t* = 12)_ – Y_i (*t* = 0)_] - [Y_c (t = 12)_ – Y_c (t = 0)_].

Where Y_i(t = 12)_ is mean SBP in the intervention villages at the end-line survey (12 months), Y_i(t = 0)_ is mean SBP in the intervention villages at baseline survey, Y_c(t = 12)_ is mean SBP in the control villages at the end-line survey (12 months); and Y_c(t = 0)_ is mean SBP in the control villages at the baseline survey.

Multi-level analysis with mixed-effects models will be used to account for inter-observer and intra-observer variations in the groups at different times (at baseline and at end-line assessments). The intervention effect size will be reported as mean differences with 95% CIs.

Similar methods of DID will be used for the analysis of secondary outcomes including changes in mean DBP, changes in the proportion of other CVD risk factors, and changes in 10-year risk for CVD event. The 10-year risk for CVD event will be estimated using the Globorisk office charts [46] specific for Tanzania. The Globorisk office chart is a simple instrument which requires age, SBP, self-reported smoking status, and gender to predict one’s 10-year risk for CVD event. For participants in the intervention, changes in 10-year risk will be assessed by comparing the risk at baseline and end-line assessment.

Missing data and loss to follow-up are major issues of concern as they may compromise inferences of any trial. Efforts will be made during trial implementation to ensure complete follow-up and minimize missing data and loss to follow-up. During analysis, we will assess the magnitude and compare the baseline characteristics of participants lost to follow-up and those with end-line assessment data to see if there is any specific pattern of missing and loss to follow-up. Since there is no single universal method for handling missing data in trials [47], it is difficult to decide a priori the best way to handle missing data for this study. The decision to exclude missing data from final analysis or use multiple imputation analysis will be made after examining the magnitude and pattern of missing data. For all analyses, a two-sided *p* value of ≤ 0.05 will considered statistically significant.

#### Qualitative data

Analysis of qualitative data from the FGDs will be done using Nvivo 9® software and manual coding. The audio recordings from FGDs will be transcribed verbatim and translated into English. Caution will be taken to ensure that no data will be lost during translation. The text data will be entered into Nvivo 9® software and analyzed for themes on perceptions about quality and acceptability of the CHWs intervention in the community.

### Handling and storage of data

Participants will be assigned unique study identification numbers for data collection. Except for consent forms, which will bear the name and signature of the participant; all other information will be collected using unique study participants’ identification numbers. All paper forms will be double-entered into a password-protected computer by trained data entry clerks. The data will be transferred daily to a secure server where the data manager will review the entered data for quality assurance and quality control. Entered paper forms will then be stored and locked at a central office, with access to the files granted only to the study’s principal investigator and key personnel. Any reports or publications or scientific presentations will not contain any participants’ identifying information.

### Dissemination of results

Sharing of the findings is an essential part for this study to have an impact on CVD prevention. To ensure that the study findings are known to the local communities, policy makers, and to the scientific community, we plan to do the following.

#### Dissemination to the local communities

The final results of this trial will be communicated in Swahili using simple and non-scientific language to the communities where the study will be conducted. The results will also be shared with the communities, through dissemination meetings, and with the Village Ward Health Committees, as well as the district authorities in Kilombero and Ulanga districts.

#### Dissemination to policy makers in Tanzania

We plan to meet and share the findings of the trial with senior personnel at the Ministry of Health, Community Development, Gender, Elderly and Children. This will be helpful in formulating appropriate policy in line with the nation-wide scale-up of the CHWs’ cadre to encourage their engagement in providing CVD health education, healthy lifestyle promotion services to the community, and to screening for CVD risk factors for early identification and referral for appropriate management.

#### Dissemination to the scientific community

We will present and discuss the results of this trial regularly at the research seminars at Muhimbili University of Health and Allied Sciences. These seminars are attended by teaching staff and postgraduate and undergraduate students. We also plan to present the findings of this study to national, regional, and international scientific conferences. Findings from this study will also be submitted for publication in peer-reviewed journals for wider dissemination.

## Discussion

This is the first large-scale randomized controlled trial investigating the effects of training CHWs and their interventions on CVD risk factors among an adult population in Tanzania. The intervention consists of home-delivered health education and healthy lifestyle promotion for the prevention of CVD risk factors. The study compares the outcomes related to knowledge and prevalence of CVD risk factors in the intervention and control villages. This study will provide evidence on the effectiveness of CHWs’ interventions on improving knowledge and the reduction of CVD risk factors among adults in Tanzania.

The primary prevention of CVDs through CHW-delivered interventions are highly required in LMICs such as Tanzania which are faced by a double burden of prevalent communicable diseases and rising NCDs, compounded by a shortage of both financial and human resources for the delivery of healthcare services. This study will be conducted among semi-urban/rural communities in Kilombero and Ulanga districts in Morogoro which are faced with increasing NCD-related mortality [8] and generally limited access to better healthcare services [48]. The selected study areas are therefore ideal for CHW-led interventions to improve knowledge, identify early those at high risk for CVD, and promote healthy lifestyle for the reduction of CVD risk factors in the community.

This study has some limitations worth mentioning. First, there is no guarantee of comparable distribution of sociodemographic characteristics such as age and sex between intervention and control villages. Therefore, the true effect of the CHW interventions may be distorted by the natural progression of the diversity in the pattern of CVD risk factors. These variations will be minimized by using multi-level analysis with random effect models. Second, the diagnosis of hypertension using blood pressure readings taken on a single visit may overestimate the true prevalence of hypertension in the population. However, using an average of three blood pressure measurements (halfway and at the end of interview) will minimize the risk of white coat hypertension. Additionally, we will repeat the measurements on a separate occasion for individuals with elevated blood pressure to confirm hypertension. The risk of contamination of the intervention will be minimized by grouping and randomizing a group of three close villages to the same intervention arm. This will ensure that for each district, intervention villages will be together but far away (approximately 20 km) from control villages. There are still chances of interaction between people in the intervention and control villages. Other limitations of the study include unmeasured residual confounding and measurement bias for anthropometric measurements.

In conclusion, this study is the first randomized trial investigating the feasibility and effectiveness of CHW home-delivered interventions for the reduction of CVD risk factors in Tanzania. The findings of this study may be beneficial in influencing policy changes to enhance home delivery of health education, screening, and healthy lifestyle promotion interventions for the reduction of CVD risk factors in communities using CHWs.

## Trial status

The trial protocol reported here is version 1.3 (20 December 2017). The study has not started recruitment. Field activities will start soon upon obtaining approval from local government authorities.

## Additional file

Additional file 1: SPIRIT 2013 Checklist: Effect of training CHWs and their interventions on CVD risk factors among adults in Morogoro, Tanzania: study protocol for cluster randomized controlled trial. (DOCX 60 kb)

## Abbreviations

BMI: Body mass index
CHWs: Community health workers
CVDs: Cardiovascular diseases
DBP: Diastolic blood pressure
DID: Difference-in-differences
DM: Diabetes mellitus
FGDs: Focus group discussions
HDSS: Health and demographic surveillance system
HICs: High-income countries
LMICs: Low- and middle-income countries
NCDs: Non-communicable diseases
SBP: Systolic blood pressure
SSA: Sub-Saharan Africa
WHO: World Health Organization
